# Postglacial migration supplements climate in determining plant species ranges in Europe

**DOI:** 10.1098/rspb.2010.2769

**Published:** 2011-05-04

**Authors:** Signe Normand, Robert E. Ricklefs, Flemming Skov, Jesper Bladt, Oliver Tackenberg, Jens-Christian Svenning

**Affiliations:** 1 Ecoinformatics and Biodiversity Group, Department of Biological Sciences, Aarhus University, Ny Munkegade 114, 8000 Aarhus C, Denmark; 2 Department of Biology, University of Missouri-Saint Louis, 1 University Boulevard, Saint Louis, MO 63121-4499, USA; 3 Department of Wildlife Ecology and Biodiversity, National Environmental Research Institute, Aarhus University, 8410 Rønde, Denmark; 4 Institute of Ecology, Evolution and Diversity, Goethe University Frankfurt, Max-von-Laue Strasse 13, 60438 Frankfurt am Main, Germany

**Keywords:** ecological niche modelling, hind-casting, ice age refugia, disequilibrium, plant species distributions, postglacial recolonization

## Abstract

The influence of dispersal limitation on species ranges remains controversial. Considering the dramatic impacts of the last glaciation in Europe, species might not have tracked climate changes through time and, as a consequence, their present-day ranges might be in disequilibrium with current climate. For 1016 European plant species, we assessed the relative importance of current climate and limited postglacial migration in determining species ranges using regression modelling and explanatory variables representing climate, and a novel species-specific hind-casting-based measure of accessibility to postglacial colonization. Climate was important for all species, while postglacial colonization also constrained the ranges of more than 50 per cent of the species. On average, climate explained five times more variation in species ranges than accessibility, but accessibility was the strongest determinant for one-sixth of the species. Accessibility was particularly important for species with limited long-distance dispersal ability, with southern glacial ranges, seed plants compared with ferns, and small-range species in southern Europe. In addition, accessibility explained one-third of the variation in species' disequilibrium with climate as measured by the realized/potential range size ratio computed with niche modelling. In conclusion, we show that although climate is the dominant broad-scale determinant of European plant species ranges, constrained dispersal plays an important supplementary role.

## 1. Introduction

The ability of species to track areas of suitable climate through time is a major source of uncertainty when predicting climate change impacts on biodiversity. Climate is generally regarded as the main determinant of species ranges at broad geographical scales [1], while the role of dispersal is poorly understood and controversial [2]. The palaeoecological record provides ample evidence that Quaternary climate changes caused dramatic shifts in species distribution [3,4]. During the Last Glacial Maximum (LGM, *ca* 21 kyr ago), most European species were restricted to latitudes south and east of the Scandinavian icecap, but post-LGM warming allowed species to expand northward. However, the degree to which species have been able to track climate during the Late glacial and postglacial periods is an old controversy [3,5,6] that is still being debated [7,8]. Some palaeoecological studies indicate migrational lags [9,10], while others suggest that species migrated fast enough to maintain equilibrium with climate [3,6], reporting rapid climate-driven community shifts or range dynamics [7,11–17]. Macroecological research suggests, in support of migration lags, that many European species are absent from climatically suitable areas and thus have ranges that are in disequilibrium with current climate [18–20]. Such absences could result from time-lagged range expansions or contemporary non-climatic factors that exclude species from certain areas (e.g. edaphic conditions and biotic interactions). Based on niche modelling and extensive naturalizations, Svenning & Skov [18] emphasized limited postglacial migration as the main reason for climatic disequilibrium of European tree species ranges, and subsequent studies documented the influence of limited dispersal on patterns of tree species richness [21], and on distribution patterns of some widespread forest plant species [22]. However, the relative importance of limited postglacial migration relative to current climate in determining species ranges more generally is currently unknown.

The degree to which species have been able to track past climate change might depend on competition with already established vegetation, non-climatic factors (e.g. soil development or human influence), dispersal ability and reproductive age [3,5]. For example, ferns might exhibit shorter lags compared with seed plants owing to their easily dispersed spores [23], while the dispersal rates of trees compared with herbs might be slower owing to their higher reproductive age. Furthermore, long-distance dispersal (LDD) is likely to influence large-scale range expansion rates [24]. While a species' local dispersal ability is often linked to its typical dispersal mode as reflected by its diaspore morphology, LDD often depends on extreme weather events or migrating animals [25,26]. Regardless, species with morphological adaptations to multiple vectors with high LDD potential should be dispersed long distances more often, and should thus experience less migrational lag than other species.

In addition to their dispersal abilities, the location of species' LGM ranges may also influence their post-LGM expansion patterns [27], particularly by affecting their access to currently climatically suitable areas. Notably, current distributions of species with larger, more northern LGM ranges are less likely to be limited by postglacial migration. During the LGM, Mediterranean and temperate species were predominantly restricted to refugia in southern Europe, although evidence also suggests the presence of some temperate species in southern parts of central and eastern Europe (e.g. [28–30]). By contrast, arctic and probably also many boreal species had wider ranges across Central and Eastern Europe [4,13,30–34], facilitating postglacial climate tracking. The ranges of these species are therefore likely to be closer to equilibrium with current climate. Alpine species in central and southern Europe may represent cold-adapted species, which for intrinsic or extrinsic reasons have failed to expand into northern Europe despite a suitable climate in the region.

In the present study, we assessed the influence of current climate and time-lagged migration following Late glacial and postglacial warming (simply referred to as postglacial migrational lag) on the ranges for more than 1000 plant species. We estimated migrational lag by how well a species' current distribution is explained by a species-specific measure of geographical variation in accessibility to colonization from its LGM range (estimated by hind-casting, cf. [32]). The few previous studies that examined the importance of accessibility used a simple generalized measure for all species [21,22]. The development of a species-specific accessibility measure is a clear improvement, because refugia and postglacial migration routes—despite some generalities—have varied idiosyncratically among species (e.g. [35]). Using logistic regression modelling, we assessed the relative importance of current climate and postglacial accessibility for each species. Our study questions were: (i) how important is accessibility relative to climate for determining European plant species ranges? (ii) does the importance of accessibility vary among species according to their dispersal ability (as represented by different life forms and their LDD vectors), postglacial geographical dispersal opportunities (as represented by climate zone associations and resulting likely LGM ranges), or range size? (iii) to what degree does accessibility explain species' disequilibrium with climate, if present? and (iv) does climate predict distribution more closely for species with large postglacial range shifts?

## 2. Material and methods

### (a) Study species and area

We considered the geographical distributions of 2728 native European plant species mapped in the Atlas Florae Europaeae (AFE) on an equal-area grid with cells of *ca* 50 × 50 km (AFE cells) (see the electronic supplementary material, appendix S1 for further details). To avoid overfitting and unstable parameter estimates in the regression modelling (see below), we assured a minimum of 10 events per parameter [36] by removing species with 65 or less presences (*n* = 1711) or 65 or less absences (*n* = 1), leaving 1016 study species.

We used different study areas for computing accessibility and range shifts and for modelling of current species distributions (see the electronic supplementary material, appendix S1). The former involved predicting the LGM range of each species and therefore required good estimates of their ecological tolerances, particularly of cold and drought. We therefore calibrated the species distribution models used for these computations using both native and naturalized occurrences across all of Europe, including the former Soviet Union (*n* = 4878 AFE cells), because this region ranges into cold and dry areas with an LGM-like climate [37]. Projections were performed for a smaller area owing to more limited geographical coverage of the LGM climate simulations. Logistic regression modelling requires reliable presence–absence data, and the analyses of current species distributions were therefore performed on their native ranges in Europe (*n* = 2276 AFE cells), excluding the former Soviet Union because of incomplete registration of species ranges there.

### (b) Climate data

Data regarding current climate were obtained from the CRU CL 2.0 dataset at a 10′ resolution (period 1961–1990 [38]). For LGM climate, we used both the Stage 3 Project simulation [39] and Laboratoire de Météorologie Dynamique's (LMDZHR) simulation [40] (*ca* 60 km resolution). To improve the representation of topoclimatic variation, these simulations were downscaled to 10′ resolution as in Svenning *et al*. [32]. From monthly values of mean temperature and precipitation, we derived three key bioclimatic variables: absolute minimum temperature of the coldest month (TMIN), growing-degree-days (GDD) and water balance (WBAL) (see the electronic supplementary material, appendix S1).

### (c) Estimating Last Glacial Maximum species ranges

Owing to incomplete sampling in the former Soviet Union, we applied two presence-only species distribution modelling (SDM) algorithms: maximum entropy species distribution (Maxent) modelling [41] and a standard rectilinear climatic envelope (Bioclim) model [42]. Maxent performs well compared with other SDM methods [41,43], but may provide narrow climatic niche estimates. Among alternative SDM methods, we chose Bioclim as an alternative because it generally provides results that are among the most divergent from Maxent [43].

The models were calibrated on species occurrences, using AFE cell means for the three bioclimatic variables, but projected onto 10′ LGM climate data (see the electronic supplementary material, appendix S2 for details on the modelling procedure). Species might not have occupied all areas predicted as climatically suitable. Optimally, the estimated LGM range for each species should be evaluated against the palaeoecological record or, alternatively, phylogeographic evidence. This information, however, is not available for all species studied here, and we only evaluated the LGM ranges for some species (see §4 and the electronic supplementary material, appendix S2). To account for possible overestimation of species LGM occurrence, we choose two approaches: (i) scoring LGM presence for all AFE cells where a given species was predicted to be present in at least one 10′ pixel (unrestricted LGM range), and (ii) defining refuge regions (e.g. Balkans, Iberian Peninsula, Italy) and then restricting the LGM range as estimated by the first approach to those regions where the species presently occurs (restricted LGM range) (see the electronic supplementary material, appendix S2).

### (d) Estimating postglacial migrational lag and range shift

**Figure 1.**
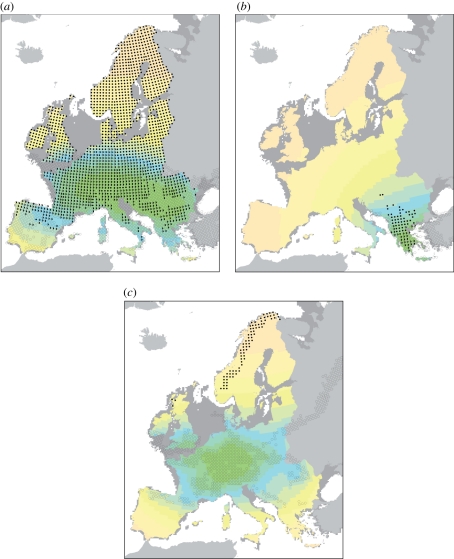
Distribution, postglacial accessibility and variation in species occurrences uniquely explained by accessibility 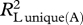 or climate 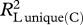 for selected species: (*a*) *Filipendula ulmaria*; 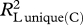 64%, 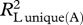 0%; (*b*) *Ranunculus psilostachys*; 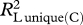 14%, 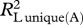 45%; (*c*) *Koenigia islandica*; 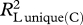 73%, 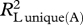 0%. Current distribution (black dots), hind-casting-based estimate of LGM distribution (empty circles), overlap between the two (half-filled circles) and accessibility to postglacial colonization from the estimated LGM range (green to orange: high to low). Maps are in the ETRS 1989 Lambert Azimuthal Equal Area projection.

If postglacial migrational lag constrains species distributions, we would expect species to be more common close to their LGM ranges (i.e. areas with high accessibility to postglacial colonization). To quantify postglacial accessibility, we used an approach that builds on Svenning & Skov [21], but improves upon it by using species-specific LGM range estimates: for each AFE cell, accessibility to postglacial colonization (ACC) was calculated by summing the inverse of the geographical distance (in km) between the given AFE cell and each of the AFE cells in the species' LGM range. Hence, the more distant an AFE cell is from the LGM range, the lower its accessibility (figure 1). ACC values calculated from the four unrestricted estimates of LGM ranges were highly correlated (average ± standard deviation (s.d.), Spearman's *ρ*: 0.95 ± 0.06; see the electronic supplementary material, appendix S2). Therefore, we conducted the regression modelling using only ACC estimates based on the restricted and unrestricted LGM ranges obtained using Maxent and the LMDZHR simulation. The results of the subsequent analyses for the two range estimates were, however, similar (see the electronic supplementary material, appendix S3), and we thus only report the analyses based on the restricted and probably more realistic LGM range estimates.

To quantify post-LGM range shifts, we calculated the latitudinal difference between the range centroids of the LGM and present distributions.

### (e) Data analysis

The relative importance of migrational lag and current climate as range determinants was evaluated by computing three logistic regression models for each species, involving either climatic predictor variables (M_C_), accessibility (M_A_) or both (M_CA_). The M_C_ and M_CA_ models included linear and quadratic terms of the climatic variables. Of the climatic variables, only WBAL needed to be square-root transformed after subtracting its original minimum to obtain absolute skewness values of less than 1.0. Skewness of ACC was generally not strong (median: 1.03, range −0.42 to 6.67). Correlations among the explanatory variables are given in the electronic supplementary material, appendix S1.

For each species, support for each model and the two range controls (current climate and accessibility) was assessed using an information-theoretic approach [44]. First, relative support for each model was assessed using Akaike's information criterion (AIC) by computing the AIC differences (*Δ*AIC) between a given model and the minimum AIC obtained in the model set. Thus, *Δ*AIC = 0 for the best model, while models with *Δ*AIC ≤ 2.0 were considered to have substantial support [44]. Second, Akaike weights (*w*) for each model indicate the probability that a given model is the best in the model set, while the sum of *w* (*W*) for models containing either accessibility or climate represent the probability that either factor was included in the best model. Furthermore, the proportion of variation in a species' occurrences explained by a given model was estimated by the likelihood ratio *R*^2^ (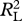), the best estimate of *R*^2^ for multiple logistic regressions [45]. Variation partitioning was used to estimate the variation uniquely explained by either climate or accessibility (hereafter 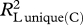 and 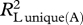), as well as the variation fraction shared (jointly explained) by climate and accessibility [46]. A negative shared fraction can sometimes occur [46]; in these cases (*n* = 157), we estimated the unique fractions by the 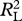 for M_C_ or M_A_, respectively.

If accessibility represented migrational lag, it should be positively related to species occurrence. We assessed this relationship by computing model-averaged parameter estimates for accessibility as *β*_MA(A)_ = *w*_CA_ × *β*_CA_ + *w*_A_ × *β*_A_, where *β* and *w* are the standardized parameter estimates for accessibility and Akaike weights in the M_CA_ and M_A_ models, respectively [44]. Because a negative *β*_MA(A)_ is not meaningful, 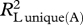 was set to zero and 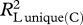 was equal to 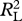 for M_C_ when this occurred. We tested whether the predicted positive accessibility relationship was supported (positive *β*_MA(A)_ and in the model with *Δ*AIC ≤ 2.0) or highly supported (positive *β*_MA(A)_ and in the best model with *W* ≥ 95%) for a majority of species using the normal approximation to the binomial test [47].

To examine whether the importance of accessibility as a range constraint varied according to life form, LDD ability or climate zone association, we tested for differences in: (i) the proportion of species with high support for accessibility using *χ*^2^-tests, and (ii) the 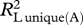 using Kruskal–Wallis rank sum and pairwise Wilcoxon tests with Bonferroni correction. The species were categorized as fern (including fern allies), annual herb, perennial herb, shrub (or woody climber) or tree. LDD ability for each species was deduced from the number of LDD vectors (i.e. anemochory, hydrochory, epizoochory, endozoochory, dysochory and hemerochory) recorded in several databases [48–50]. Each species was assigned to the climate zone that was most prevalent within its range (see the electronic supplementary material, appendix S1).

The degree to which species' ranges are in equilibrium with current climate has been measured as the ratio between the species realized (observed) distribution and potential distribution estimated with bioclimatic envelope modelling (range filling *sensu* [18]). We examined the degree to which the constraining effect of postglacial accessibility explains species' disequilibrium with current climate by relating 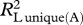 to range filling as computed in Svenning & Skov [18].

Logistic regressions, Kruskal–Wallis rank sum and pairwise Wilcoxon tests were performed in R 2.6.1 [51]. Loess regressions were fit using S-PLUS 7.0; *χ*^2^-tests were performed in SPSS 16.0.0.

## 3. Results

The model that included both climatic predictors and accessibility was the best for the vast majority (91%) of the 1016 investigated species, and it had substantial support for the remaining species for which the best model included climate only (see the electronic supplementary material, appendix S3). There was generally 100 per cent support for including accessibility and climate in the best model (table 1). Models for the majority of the species (65%) had a positive *β*_MA(A)_ (one-tailed binomial test, *p* < 0.001), and more than half (55%) additionally included accessibility with at least 95 per cent support (one-tailed binomial test, *p* < 0.01).

**Table 1.** Relative importance of climate and accessibility for species occurrences. [*W*, average (±s.d.) and median (minimum; maximum) summed Akaike weights for climate and accessibility. 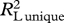, average (±s.d.) and median (minimum; maximum) proportion of variation in species occurrences uniquely explained by climate or accessibility after controlling for the other factor. The values were either calculated across all species (*n* = 1016) or only for species with a positive model-averaged parameter accessibility coefficient (positive *β*_MA(A)_, *n* = 655).]

	all		positive *β*_MA(A)_	
	*W* (%)	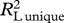 (%)	*W* (%)	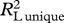 (%)
climate	100 ± 0.3	28.5 ± 16.3	100 ± 0.4	22.4 ± 12.8
	100 (91.2; 100)	25.8 (1.5; 80.9)	100 (91.2; 100)	20.5 (1.5; 64.1)
accessibility	91.4 ± 20.5	5.8 ± 8.9	93.2 ± 18.5	8.9 ± 9.8
	100 (26.9; 100)	0.7 (0; 51)	100 (26.9; 100)	5.4 (0; 51)

**Figure 2.**
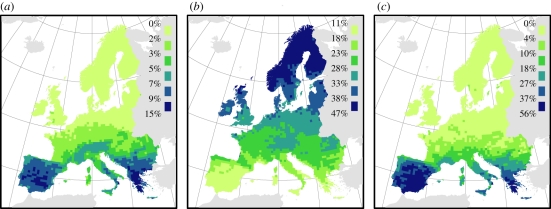
Geographical patterns in the importance of accessibility and climate for species occurrences across Europe. Average variation in species occurrences uniquely explained by (*a*) accessibility 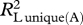, or (*b*) climate 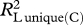. (*c*) Percentage of species with 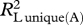 > 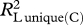. All values were calculated across species with a positive model-averaged accessibility coefficient (*n* = 655) in each *ca* 50 × 50 km AFE cell. Maps are in the ETRS 1989 Lambert Azimuthal Equal Area projection.

On average (±s.d.), climate uniquely explained five times more variation in species distributions than in accessibility (table 1). However, accessibility explained more variation in species occurrences than climate for 16 per cent of all species. The importance of climate and accessibility exhibited clear geographical patterns: 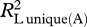 and 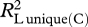 increased towards southern and northern Europe, respectively (figure 2). In southern Europe, 18–56% of the species per AFE cell had a higher 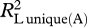 than 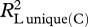 (figure 2). Species' occurrences were generally best explained in northern Europe (see the electronic supplementary material, appendix S3).

**Figure 3.**
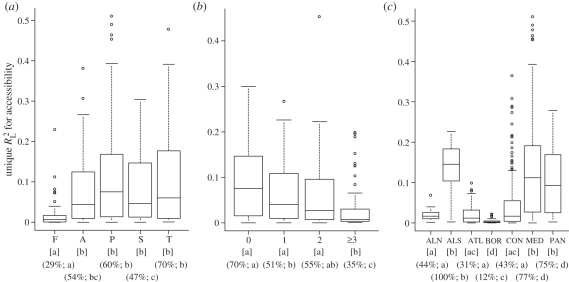
Variation in species occurrences uniquely explained by accessibility for species with different (*a*) life forms (F, fern; A, annual herb; P, perennial herb; S, shrub; T, tree), *n* = 595; (*b*) long-distance dispersal (LDD) potential (measured as the number of LDD vectors), *n* = 346; and (*c*) climate-zone associations (ALN, northern-alpine; ALS, southern-alpine; ATL, Atlantic; BOR, boreal; CON, continental; MED, Mediterranean; PAN, Pannonian) *n* = 655 (shown for species with positive model-averaged accessibility coefficients, *β*_MA(A)_). In squared parentheses, identical letters indicate no significant difference between groups (*p* < 0.05; Mann–Whitney *U*-test, significance levels adjusted using Bonferroni correction). In round parentheses, percentage of all species in a given group with a positive *β*_MA(A)_, and summed Akaike weights for accessibility of ≥95%; identical letters indicate no significant differences in percentages among groups (tested using *χ*^2^-tests, see the electronic supplementary material, appendix S4).

As anticipated, 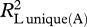 was lower for ferns than for other life forms (figure 3*a*). Accordingly, accessibility had high support (positive *β*_MA(A)_ and *W* ≥ 95%) for only 29 per cent of all ferns, while this was the case for 70 per cent of trees (figure 3*a* and the electronic supplementary material, appendix S4). Also as predicted, 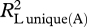 decreased the more LDD vectors a species had, and accessibility was only supported for 35 per cent of the species with more than three LDD vectors (figure 3*b* and the electronic supplementary material, appendix S4). The variation in support for accessibility among species in different climate zones also followed expectations: accessibility was unimportant for boreal species; of low, but significantly higher importance for northern-alpine and temperate species (Atlantic and continental zone) and of much higher importance for species of low-latitude climate zones (figure 3*c* and the electronic supplementary material, appendix S4). Also as expected, climate was of low importance for Mediterranean species, of higher importance for southern-alpine, temperate and, in particular, boreal species and of much higher importance for northern-alpine species (see the electronic supplementary material, appendix S4).

**Figure 4.**
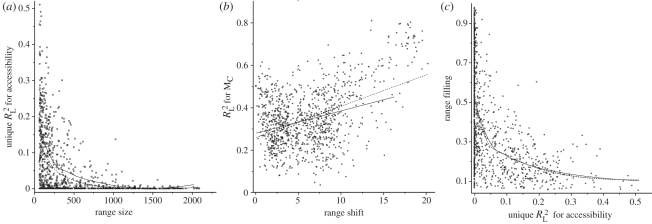
Relationship between the importance of accessibility for species occurrences and (*a*) species current range size, (*b*) estimated range shift since LGM, and (*c*) range filling. Unique 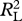 for accessibility represent the variation in species occurrences uniquely explained by accessibility after controlling for the effect of climate (shown for all species, *n* = 1016). Linear and Gaussian local (loess, fitted with span = 0.75 and a quadratic term) regressions were fitted either for all species or only for species with a positive model-averaged accessibility coefficient (*n* = 655). (*a*) Dashed line with circles, all (loess: *R*^2^ 0.23); solid line, *β*_MA(A)_ > 0 (loess: *R*^2^ 0.29); (*b*) dashed line with circles, all (linear: *R*^2^ 0.20); solid line, *β*_MA(A)_ > 0 (linear: *R*^2^ 0.07); (*c*) dashed line with circles, all (loess: *R*^2^ 0.35); solid line, *β*_MA(A)_ > 0 (loess: *R*^2^ 0.37).

The importance of accessibility was higher for species with small ranges (figure 4*a*), while no clear trend was observed for climate (see the electronic supplementary material, appendix S3). Climate, however, increased in importance with the estimated range shift since the LGM (figure 4*b*). The relationship of range filling with 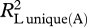 exhibited a lower triangular form: where accessibility was important, range filling was low, while where accessibility was unimportant, range filling was either high or low (figure 4*c*). Accessibility explained 35 per cent of the variation in range filling across all species.

## 4. Discussion

We found evidence that European plant species ranges are strongly shaped by current climate, but that postglacial migrational lag constitutes an additional constraint for more than 50 per cent of species, being more important than climate for 16 per cent. Hence, our results support the view that climate is the main determinant of species ranges, with many species having a high degree of range equilibrium with current climate [3,6,13]. At the same time, our results also show that more than half the species have ranges that are constrained by accessibility to postglacial recolonization and thus have not fully expanded in response to postglacial warming [5,9,10,22], showing at least partial disequilibrium with climate.

Differences in migrational lag among species have been attributed to differences in intrinsic dispersal abilities, soil development, competition with established vegetation during migration, geographical barriers, human habitat fragmentation and LGM range location [3,5]. We did indeed find that the importance of accessibility varied according to life forms, dispersal ability and LGM location of species' preferred climate zone. These patterns, discussed below, support the idea that our measurement of postglacial accessibility reflects limited postglacial migration. However, we cannot exclude contributions of other above-mentioned factors to the observed accessibility relationships.

Several patterns linked the estimated strength of postglacial accessibility to autoecological dispersal ability. LDD is regarded as an important factor in determining broad-scale distribution patterns, including range shifts under climate change [24,26]; therefore, species with more vectors having high LDD potential should have greater probability of colonizing across long distances. In agreement with this expectation, we found that the ranges of species with three or more LDD vectors are least constrained by postglacial migration. Furthermore, accessibility is of little importance for ferns compared with other life forms, in accordance with their minute, easily dispersed spores (figure 3). Previous studies of fern distributions in other regions similarly concluded that they are less dispersal-limited than seed plants [23,52] (but cf. [22]).

Differences in the importance of postglacial accessibility varied as expected among species associated with different climate zones; i.e. in accordance with the latitudinal location of their LGM distributions. The scarce evidence for migrational lag among boreal species agrees with Svenning *et al*. [22], and was expected, given the increasing evidence that boreal species survived the LGM in central and/or eastern Europe [13,30–33,53] with easy post-LGM access to northern Europe. *Picea abie*s, *Pinus sylvestris*, *Betula pendula* and *Betula pubescens* exemplify species for which palaeoecological evidence indicates northern LGM occurrences [13,34,53,54] and for which we found accessibility to be unimportant (see the electronic supplementary material, appendix S2). By contrast, accessibility was found to supplement climate in explaining ranges of the temperate tree species *Fagus sylvatica* and *Abies alba* for which our modelling as well as palaeoecological data indicate that LGM occurrences were mainly limited to southern central and southern Europe [14,29,30] (see the electronic supplementary material, appendix S2). As will be the case for many species, the exact postglacial expansion of *F. sylvatica* was more geographically complex than implied by our accessibility measure [29], most probably leading to an underestimation of the importance of accessibility. Previous studies mainly concentrated on trees, but our analyses suggest that these findings can be generalized across life forms, at least for seed plants (figure 3). For example, our modelling suggests that the widespread herb *Filipendula ulmaria* had a wide, relatively northern LGM distribution (figure 1), and that its current distribution is controlled by climate, but is unconstrained by limited postglacial migration. Accordingly, fossil evidence documents its rapid Late glacial colonization of central and northern Europe, and suggests *in situ* LGM survival as far north as southern England [55]. *Ranunculus acris*, *Trollius europaeus* and *Rumex acetosella* provide similar examples [55,56] (see the electronic supplementary material, appendix S2).

Species of the low-latitude climate zones survived the LGM within southern Europe [57]. In line with previous studies of diversity patterns in trees [58], we found that past climate change poses a strong constraint on the distribution of these southern species. Notably, we found that accessibility is more important than climate for 20–60% of the species in each Mediterranean grid cell (figure 2); e.g. *Ranunculus psilostachys* (figure 1), *Platanus orientalis* and *Ostrya carpinifolia* (see the electronic supplementary material, appendix S2). In contrast to Mediterranean species, the other southern species (alpine and Pannonian) were probably forced to shift and/or contract their ranges during the post-LGM warming [4,31]. Correspondingly, climate is more important for these species, although their ranges are clearly also constrained by limited postglacial migration (figure 3*c* and the electronic supplementary material, appendix S4). In the case of alpine species, constrained migration explains their failure to colonize climatically suitable areas in northern Europe; e.g. *Pritzelago alpina* (electronic supplementary material, appendix S2), for which phylogeographic evidence suggests a broader LGM range [59]. *Pulsatilla alpina* provides a similar example (see the electronic supplementary material, appendix S2). Here, these species present a striking contrast to arctic species, which also had wider LGM ranges, but successfully colonized northern Europe afterwards. Climate is a strong range determinant for arctic species, while accessibility has little importance. *Koenigia islandica* is a good example of such a species; fossil evidence [4,55], as well as our modelling (figure 1), indicates that it was distributed in central Europe during the LGM, but retracted to northern latitudes during the Holocene. Several arctic-alpine species (*Betula nana*, *Arabis alpina*, *Dryas octopetala*, *Salix herbacea*) provide similar examples [4,13,55,60,61] (see the electronic supplementary material, appendix S2).

Echoing the climate zone differences, the importance of climate was highest in northern Europe, while accessibility was most important and explained more than climate for *ca* 20–60% of the species in southern Europe (figure 2), suggesting that northern species generally are more in equilibrium with climate, while the restriction of many species to southern Europe at least partially reflects postglacial dispersal limitation. However, it is important to note that the importance of accessibility in southern Europe probably to some extent also reflects the geographical heterogeneity of the region, notably its mountainous barriers and division into multiple peninsulas [62], i.e. more long-term dispersal limitation. Although our analyses suggest that most Mediterranean species are dispersal limited, others such as *Quercus ilex* are expanding northward in response to recent climate warming and their northern range limits might thus mainly be climatically limited [63] (see the electronic supplementary material, appendix S2). Furthermore, European landscapes have been transformed by human activities for millennia, especially in the Mediterranean. These activities might have increased dispersal limitation for some species (cf. [64]), while other species (e.g. *R. acetosella*) have probably benefitted from them [55] (see the electronic supplementary material, appendix S2).

Providing further evidence that postglacial dispersal dynamics influence the relative importance of accessibility and current climate, we found that the importance of climate increased with increasing post-LGM range shift (figure 4). This suggests that species which can expand or shift ranges have more climatically controlled distributions. In addition, we also found that species with strong accessibility relationships filled little of their potential range and that accessibility explained one-third of the variation in species disequilibrium with climate (figure 4), supporting the hypothesis that migrational lag is responsible for the absence of some species from climatically suitable sites [18]. The unexplained variation in range filling, as well as species with low range filling for which we found no importance of accessibility (figure 4), draw attention to other range-limiting factors that might exclude species from suitable areas (e.g. edaphic conditions, habitat availability and biotic interactions).

The variation in species occurrences uniquely explained by accessibility increased with decreasing range size (figure 4*a*). With this result in mind, it is important to note that rare species were under represented in our analyses: approximately 65 per cent of the species for which distribution data are available in AFE had a range size of 65 or less AFE cells, and were therefore excluded from the analysis. Given the relationship depicted in figure 4*a*, we might expect these species to be even more limited by migrational lag than those common enough to be analysed.

Uncertainties related to SDM and past climate simulations should be kept in mind when interpreting our results [32,65] (further discussed in the electronic supplementary material, appendix S2). Most importantly, SDM relies on the assumption that a species climate niche can be estimated from the climate conditions where the species currently occurs (equilibrium assumption [42]). Some disequilibrium with climate does not, however, preclude achieving reliable niche estimates. The quality of the niche estimates and importance of disequilibrium in geographical space depend on the uniqueness of the environmental conditions at the sites from which the species is absent. We took several steps in order to reduce these uncertainties (see §2 and electronic supplementary material, appendix S2) and our results were consistent across modelling methods and LGM climate simulations used. Additional sources of uncertainty that could not be integrated in our modelling are the influence of inter-annual climate variability on species distributions [66], and lower carbon dioxide (CO_2_) levels and increased wind speed at the LGM [32]. Potential changes in inter-annual variability in the past could have allowed species to occupy more or less habitat than estimated with our models, while lower CO_2_ and increased wind speed might have increased vegetation openness [32].

Using the currently most comprehensive dataset on European plant species ranges and species-specific spatially explicit measures of accessibility to colonization from estimated glacial ranges, we here provide an assessment of the relative importance of climate and limited migration for the ranges of a continental flora. We found current climate to be the main range determinant, but time-lagged migration following post-LGM warming constitutes an additional constraint for many species, especially seed plants, species with small southern ranges and those with low LDD ability and southern glacial ranges. The importance of climate agrees with widely accepted hypotheses regarding macroscale determinants of species ranges [1,22]. The general support for migrational lag, however, is of great importance as the role of dispersal as a broad-scale determinant of species ranges is controversial (e.g. [2]) and provides insight into the impact of future climate change on species distributions. Our results suggest that more than 50 per cent of European plant species have been unable to fully respond to the post-LGM warming and might therefore not be able to efficiently track climatic warming of a comparable magnitude over the next 90–100 years.
